# Interleukin‐38 alleviates cardiac remodelling after myocardial infarction

**DOI:** 10.1111/jcmm.14741

**Published:** 2019-11-20

**Authors:** Yuzhen Wei, Yin Lan, Yucheng Zhong, Kunwu Yu, Wenbin Xu, Ruirui Zhu, Haitao Sun, Yan Ding, Yue Wang, Qiutang Zeng

**Affiliations:** ^1^ Institute of Cardiology Union Hospital TongJi Medical College Huahzong University of Science and Technology Wuhan China

**Keywords:** cardiac remodelling, inflammation, interleukin‐38, myocardial infarction

## Abstract

Excessive immune‐mediated inflammatory reaction plays a deleterious role in ventricular remodelling after myocardial infarction (MI). Interleukin (IL)‐38 is a newly characterized cytokine of the IL‐1 family and has been reported to exert a protective effect in some autoimmune diseases. However, its role in cardiac remodelling post‐MI remains unknown. In this study, we found that the expression of IL‐38 was increased in infarcted heart after MI induced in C57BL/6 mice by permanent ligation of the left anterior descending artery. In addition, our data showed that ventricular remodelling after MI was significantly ameliorated after recombinant IL‐38 injection in mice. This amelioration was demonstrated by better cardiac function, restricted inflammatory response, attenuated myocardial injury and decreased myocardial fibrosis. Our results in vitro revealed that IL‐38 affects the phenotype of dendritic cells (DCs) and IL‐38 plus troponin I (TNI)‐treated tolerogenic DCs dampened adaptive immune response when co‐cultured with CD4^+^T cells. In conclusion, IL‐38 plays a protective effect in ventricular remodelling post‐MI, one possibility by influencing DCs to attenuate inflammatory response. Therefore, targeting IL‐38 may hold a new therapeutic potential in treating MI.

## 1 INTRODUCTION

Acute myocardial infarction (AMI) is one of the leading causes of death worldwide associated with profound systemic inflammatory response.^1^, ^2^, ^3^ Though early coronary reperfusion strategies could dramatically improve survival rates in AMI patients, reducing the risk of heart failure followed by MI is all‐important. There is no doubt that inflammation and immune responses are particularly active in ventricular remodelling post‐infarction and eventual host outcome.^4^, ^5^, ^6^ Growing evidence has indicated that effectively repression and containment of inflammation after MI are prerequisites for adequate cardiac healing.^7^, ^8^, ^9^ Experimental studies from our laboratory have demonstrated that Treg cells can inhibit inflammation to protect against adverse ventricular remodelling and contribute to improve cardiac function after MI.^10^ Moreover, previous report in our team indicated that IL‐37 could induce immune tolerance to ameliorate myocardial injury.^11^ Thus, it is becoming increasingly apparent that timely suppression of excessive inflammation after MI are necessary for ensuring optimal formation of a supportive scar in the infarcted area and preventing malignant ventricular remodelling.^8^

IL‐38 is located in the IL‐1 family cluster on chromosome 2 and encoded by IL1F10.^12^, ^13^ As with other members of IL‐1 family, it lacks a signal peptide and caspase‐1 consensus cleavage site. IL‐38 plays biological effects through acting on IL‐1 receptor antagonist (IL‐1Ra) and IL‐36 receptor antagonist (IL‐36Ra).^13^, ^14^, ^15^ Many studies have been reported that IL‐38 gene polymorphisms are associated with many inflammatory diseases, such as rheumatoid arthritis, psoriatic arthritis and ankylosing spondylitis.^16^, ^17^, ^18^ In addition, the expression levels of IL‐17 and IL‐22 can be suppressed by IL‐38 in candida albicans‐stimulated peripheral blood mononuclear cells (PBMCs).^14^ Moreover, depletion of IL‐38 in cultured apoptotic macrophages exacerbated the expression of IL‐6 and IL‐8.^19^ We have previously shown that plasma IL‐38 level in ST‐segment elevation myocardial infarction (STEMI) patients was positively correlated with CRP, cTNI and NT‐proBNP, but was weakly negatively correlated with left ventricular ejection fraction (LVEF).^20^ Our data indicated that IL‐38 appears to be a potentially novel biomarker for patients with STEMI. However, its exact biological effect in MI needs to be further elucidated.

In this study, we aim to clarify the effect and mechanism of IL‐38 in post‐MI remodelling. Our results indicated that rIL‐38 could ameliorate cardiac remodelling and improve cardiac function in post‐MI mice. In addition, IL‐38 imposed a regulatory phenotype on DCs and inhibited inflammatory factors secretion in vitro. Our data indicated that IL‐38 could induce a tolerogenic immune response and ameliorate post‐MI remodelling in mice.

## 2 MATERIALS AND METHODS

### 2.1 Animals

Male C57BL/6 mice used in this study were purchased from Beijing HFK Bioscience Co., Ltd (Beijing, China) and maintained in Tongji Medical College Animal Care Facility on a chow diet according to institutional guidelines. Experiments involving mice (per cage n = 5‐6) were given with normal food and water prior to experiments and kept under standard animal room conditions (temperature, 21±1˚C; humidity, 50‐60%; 0.03% CO2; 12 hours for light and 12 hours for dark). All studies were approved by the Animal Experimentation Ethics Committee of Huazhong University of Science and Technology, and the experimental methods were performed in accordance with the approved guidelines.

#### 2.1.1 Surgical protocol

MI model was induced by permanently ligating the left anterior descending (LAD) coronary artery. In brief, after anaesthesia with intraperitoneal injection of sodium pentobarbital (60 mg/kg, 1%, 5 μl/g), mice were intubated and ventilated through a rodent respirator. Adequate anaesthesia was assured by the absence of reflexes prior to surgery. Chest was opened in the third or fourth intercostal space and LAD coronary artery of the heart was ligated with a 7‐0 prolene suture. Mice in the sham‐operated group were subjected to identical operation, but without the LAD coronary artery ligation.

#### 2.1.2 Treatment and groups

C57BL/6 mice were subjected to sham surgery or LAD ligation and analysed on specific time‐point post‐MI. To elucidate the causative role of IL‐38, C57BL/6 mice were randomly divided into one of three groups and relevant experimental data were analysed at different time‐points, 1 ,3,7,14 or 28 days post‐MI. The groups were as follows: (i) sham group, in which C57BL/6 mice underwent sham operation. (ii) MI+IL‐38 group, the recombinant mouse IL‐38 treatment group; and (iii) MI+PBS group, the phosphate‐buffered saline (PBS) treatment group, in which the mice were injected in traperitoneally with 0.5 μg of rIL‐38 (Adipogen AG, Liestal, Switzerland) diluted in 200 μL PBS or with 200 μl PBS only, respectively, after MI. In addition to the above treatment, 0.5 μg of rIL‐38 diluted in 200 μL PBS or 200 μL PBS was injected in traperitoneally twice per week until the designated time‐point after surgery. Each experiment was performed a minimum of three times.

### 2.2 Echocardiography

The cardiac function of mice at one and four weeks post‐MI was evaluated non‐invasively by echocardiography performed with Vevo1100 (Visualsonics, Toronto, Canada) equipped with a 30 MHz transducer‐phased‐array transducer. Mice were anaesthetized with sodium pentobarbital and two‐dimensional echocardiographic views of the mid‐ventricular short axis and parasternal long axes were obtained. Left ventricular end‐diastolic diameter (LVEDD), left ventricular end‐systolic diameter (LVESD), ejection fraction (EF) and fractional shortening (FS) were calculated from the digital images using a standard formula as previously described.^21^ The sonographer was blinded to the randomization of mice.

### 2.3 Cell culture

#### 2.3.1 Dendritic cell

Bone marrow–derived dendritic cells (BMDCs) were generated with granulocyte‐macrophage colony‐stimulating factor (GM‐CSF) (40 ng/mL) and IL‐4 (20 ng/mL). 6‐week‐old male C57BL/6 mice were used to acquire bone marrow in this study. Bone marrow cells were depleted of red blood cells and washed with phosphate‐buffered saline (PBS), then resuspended at a density of 2 × 10^6^ cells/mL in complete culture medium (RPMI‐1640 suspended with 10% heat‐inactivated foetal calf serum, GIBCO, Carlsbad, CA) with 40 ng/mL recombinant mouse GM‐CSF (Peprotech, Rocky Hill, NJ) and 20 ng/mL IL‐4 (Peprotech) at 37°C with 5.0% CO2. Half of the culture medium was replaced with the same concentrations of GM‐CSF and IL‐4 every two days. Immature dendritic cells (imDCs) were obtained after 6‐8 days of culture. Magnetic cell‐sorting kit of CD11c (Miltenyi Biotec, Auburn, CA) was used to purify DCs from the differentiated bone marrow cells. Different DC subsets were treated as follows: (i) imDCs: No additional incubation; (ii) mDCs: CD11c^+^ imDCs were incubated with 100 ng/mL lipopolysaccharide (LPS) for 24 hours; (iii) IL‐38‐LPS‐DCs: CD11c^+^ imDCs were incubated with 100 ng/mL LPS and 50 ng/mL IL‐38. All the DCs were incubated in complete culture medium.

#### 2.3.2 Preparation of splenic CD4^+^cells

Spleens were removed from anaesthetized mice, mushed and then passed through a 100‐µm nylon mesh in PBS to remove connective tissue. Erythrocytes were excluded and the splenic mononuclear cells were collected with lymphocyte separation fluid (MP Biomedicals, USA).^22^ For the separation of CD4^+^cells, magnetic cell‐sorting kit of CD4 (Miltenyi Biotec, Auburn, CA) was used to purify CD4^+^cells from the differentiated splenic cells according to the manufacturer’s instructions.

#### 2.3.3 DC/T‐cell co‐culture

imDCs, IL‐38‐ tDC or mDCs (2 × 10^5^ cells/mL) were co‐cultured with CD4^+^ T cells (isolated using a CD4 MicroBead mouse kit; Miltenyi Biotec) (1 × 10^6^ cells/mL) from splenocytes of male C57BL/6 mice. The mixed cells were cultured in a cell incubator (37°C with 5.0% CO2) for 72 hours at in 2 mL RPMI 1640 supplemented with 10% foetal calf serum (FCS). The supernatant was collected for cytokine detection using enzyme‐linked immunosorbent assay (ELISA) according to the manufacturer’s instructions, and the cells were collected for T cells analysis by flow cytometry. Methods to generate different DC subsets: (i) imDCs: No additional incubation; (ii) mDCs: CD11c^+^ imDCs were incubated with 100 ng/mL lipopolysaccharide (LPS) for 24 hours; (iii) IL‐38‐tDC: CD11c^+^ imDCs were incubated with 50 ng/mL IL‐38 , 1 μg/mL TNI and 10 ng/mL LPS for 4 hours.

#### 2.3.4 Cardiomyocytes

Cardiomyocytes required for in vitro experiments were isolated from hearts of 1‐ to 3‐day‐old mouse. Hearts were digested in 0.05% Trypsin‐EDTA (Gibco) for about 30 minutes on ice followed by serial digestions in collagenase type II (Worthington) at 37˚C and pre‐plated twice in T75 culture flasks (Sarstedt) for removing fibroblasts. Cardiomyocytes were kept at in a cell incubator (37°C with 5.0% CO2) in Dulbecco's modified Eagle's medium (DMEM, Gibco) supplemented with 15% FCS, 1% penicillin (P, 100 U/mL, Gibco), 1% streptomycin (S, 100 µg/mL, Gibco) and 5‐bromo‐2‐deoxyuridine (BrdU, 100 μmol/L, Sigma) for 36 hours. FCS‐free DMEM will replace the medium prior to treatments for 4 hours. The different subsets were treated (i) with no additional incubation; (ii) with 300µmol/L H_2_O_2_ (Sigma); and (iii) with 300 µmol/L H_2_O_2_ and 50 ng/mL IL‐38.

#### 2.3.5 Isolation of infiltrating DCs from the infarcted heart

On the indicated days after the operation, mice were deeply anaesthetized. Mice hearts were obtained after intracardially perfusing with PBS to remove blood cells before euthanasia. Infarcted heart tissue was acquired after dissecting using fine scissors, and minced infarcted heart tissue was enzymatically digested at 37°C with a cocktail of type II collagenase (Roche Diagnostics; 1 mg/mL in HEPES buffer). Isolated cell suspensions were filtered with a 100‐μm cell strainer after digestion, then suspended in RPMI‐1640 containing 3 % FCS. Cardiac infiltrating DCs were isolated through density gradient centrifugation. For the separation of dendritic cells, cells were incubated with CD45‐PE‐cy7, CD11b‐FITC and CD11c‐APC for 30 minutes.

### 2.4 Apoptosis assay

Myocardial apoptosis was detecting by terminal deoxynucleotidyl transferase dUTP nick‐end labelling (TUNEL) staining using in situ Cell Death Detection kit (Roche Diagnostics GmbH, Mannheim, Germany) according to the manufacturer's instructions. The sections were co‐stained with anti‐sarcomeric actin antibody (Sigma‐Aldrich) to specifically mark the cardiomyocytes and then stained with the secondary antibody (tetramethylrhodamine goat anti‐mouse antibody). Total cell nuclei were stained with 4,6‐diamidino‐2‐phenylindole (DAPI). More than 5 fields in >3 different sections/animals were examined by a technician who was not informed about treatment groups, in a blinded fashion.

### 2.5 Cytokines detection

Levels of TNF‐α, IL‐10, IL‐22, IL‐23, IFN‐γ, IL‐6 and IL‐17 in cell culture supernatant were measured by an enzyme‐linked immunosorbent assay (ELISA) using corresponding kit (R&D Systems, USA) according to the manufacturer's instructions. The intra‐assay and inter‐assay variation coefficients for all ELISA were <10%.

### 2.6 Real‐time PCR analysis

Total RNA was extracted from cultured cells or tissues using Trizol (Invitrogen, Carlsbad, CA) and converted into cDNA using the PrimeScript RT reagent kit (Takara Biotechnology, Dalian, China). SYBR Green Master Mix (Takara Biotechnology, Dalian, China) was used to quantify mRNA levels of target genes with an Applied Biosystems 7500 Real‐Time PCR system (BIO‐RAD, Singapore). mRNA expression level of each sample was normalized to that of GAPDH. Primer sequences used in this study are listed in Table 1.

**Table 1:** Primer sequences for RT‐PCR

Gene	Forward primer	Reverse primer
FOXP3	5′‐TCAAAGAGCCCTCACAACCAGCTA‐3′	3′‐TTTGAAGGTTCCAGTGCTGTTGC‐5′
TNF‐α	5′‐TGCCTCAGCCTCTTCTCATT‐3′	3′‐GCTTGGTGGTTTGCTACGAC‐5′
IL‐10	5′‐CCTGCTCTTACTGGCTGGAG‐3′	3′‐TGTCCAGCTGGTCCTTCTTT‐5′
TGF‐β1	5′‐TGCTTCAGCTCCACAGAGAA ‐3′	3′‐TGGTTGTAGAGGGCAAGGAC ‐5′
IFN‐γ	5′‐ATGAACGCTACACACTGCATC‐3′	3′‐CCATCCTTTTGCCAGTTCCTC‐5′
IL‐38	5′‐TCAAGGATGCACATCAAAAGGC‐3′	3′‐AGGCAGCAACTTCCTCCCT‐5′
IL‐17A	5′‐TGTGAAGGTCAACCTCAAAGTCT‐3′	3′‐GAGGGATATCTATCAGGGTCTTCAT‐5′
IL‐6	5′‐CCGGAGAGGAGACTTCACAG ‐3′	3′‐TCCACGATTTCCCAGAGAAC‐5′
IL‐1β	5′‐CTGTGACTCGTGGGATGATG‐3′	3′‐GGGATTTTGTCGTTGCTTGT‐5′
IL‐23	5′‐ATGCTGGATTGCAGAGCAGTA‐3′	3′‐ACGGGGCACATTATTTTTAGTCT‐5′
IDO	5′‐CAGCTTCTCCTGCAATCAAAGCA‐3′	3′‐TGCGAGGTGGAACTTTCTCACAGA‐5′
GAPDH	5′‐GTGCTGAGTATGTCGTGGAG‐3′	3′‐GTCTTCTGAGTGGCAGTGAT‐5′

### 2.7 Western blot analysis

Total protein was extracted from heart tissues using RIPA lysis buffer (Beyotime) and quantified using a BCA protein assay kit (Pierce Biotechnology Inc). The following primary antibodies were used: anti‐rat GAPDH (Immunoway, USA) and anti‐mouse IL‐38 (R&D Systems, USA). Protein samples were separated on 10% SDS‐PAGE and transferred to polyvinylidene difluoride membranes. After being blocked with 5% defatted milk for 1‐2 hours at room temperature, the membranes were incubated with the appropriate primary antibodies and incubated at 4°C overnight, followed by incubation with an HRP‐conjugated secondary antibody. The specific bands were visualized using the Super ECL reagent (Thermo Scientific). ImageLab 3.0 software (Bio‐Rad Laboratories, Hercules, CA) was used for analysis.

### 2.8 Flow cytometry

For Treg analysis, the isolated cells or cultured cells were hybridized with anti‐CD4‐FITC and anti‐CD25‐APC (both from eBioscience, San Diego, CA) for about 30 minutes at 4°C and then stained with anti‐Foxp3‐PE (eBioscience) after fixation and permeabilization following the instructions. For analysis of the characterization of cultured DCs, the cells were stained with CD11c‐APC (BD Biosciences), MHC‐II‐FITC (BD Biosciences), CD40‐PE (eBioscience) or CD86‐PE (eBioscience) for 30 minutes. FACSCalibur (BD Immunocytometry Systems) and Flowjo software (TreestarInc) were used for cell detection and data analysis.

### 2.9 Immunohistochemistry and immunofluorescence

Selected heart tissues were embedded in paraffin and used for immunohistochemical studies. For the assessments of inflammatory cell areas in damaged heart, heart sections (5 μm) were stained with haematoxylin‐eosin (HE). For the analysis of collagen volume fraction, heart sections(5 μm) were stained with Masson's trichrome. The sections were incubated with primary anti‐myeloperoxidase IL‐38 (Abcam, ab180898), (MPO; Abbiotec, UK) and CD68 (eBioscience) at 4°C overnight, followed by respective secondary antibodies for 1 hour at room temperature. The numbers of MPO^+^ neutrophils and mouse CD68^+^ macrophages were assessed by counting the total cell numbers in the infarcted and border areas in 10 randomly chosen fields in each section. The microscope (BX51, Olympus, Japan) was used to observe pathological change areas. Image‐Pro Plus6.703 software (Media Cybernetics) was used for calculation of fibrosis areas and the statistical analysis. To analyse IL‐38‐expressing cells in infarcted heart, the sections were stained with specific antibodies against CD68 (1:100, Santa Cruz Biotechnology, USA) and α‐actinin, (1:100, Abcam, UK) to identify macrophages and cardiomyocytes.

### 2.10 Statistical analysis

All data are shown as means ± SEM. Differences between 2 groups and among multiple comparisons were analysed respectively using unpaired Student's test and one‐way ANOVA, followed by Bonferroni's test. Survival rate was analysed by Kaplan‐Meier survival analysis and compared by the log‐rank test. GraphPad Prism 6.0 (GraphPad Software, Inc, CA) was used for all the analyses, and *P* < .05 was considered statistically significant.

## 3 RESULTS

### 3.1 The expression of IL‐38 is highly elevated after MI

**Figure 1:**
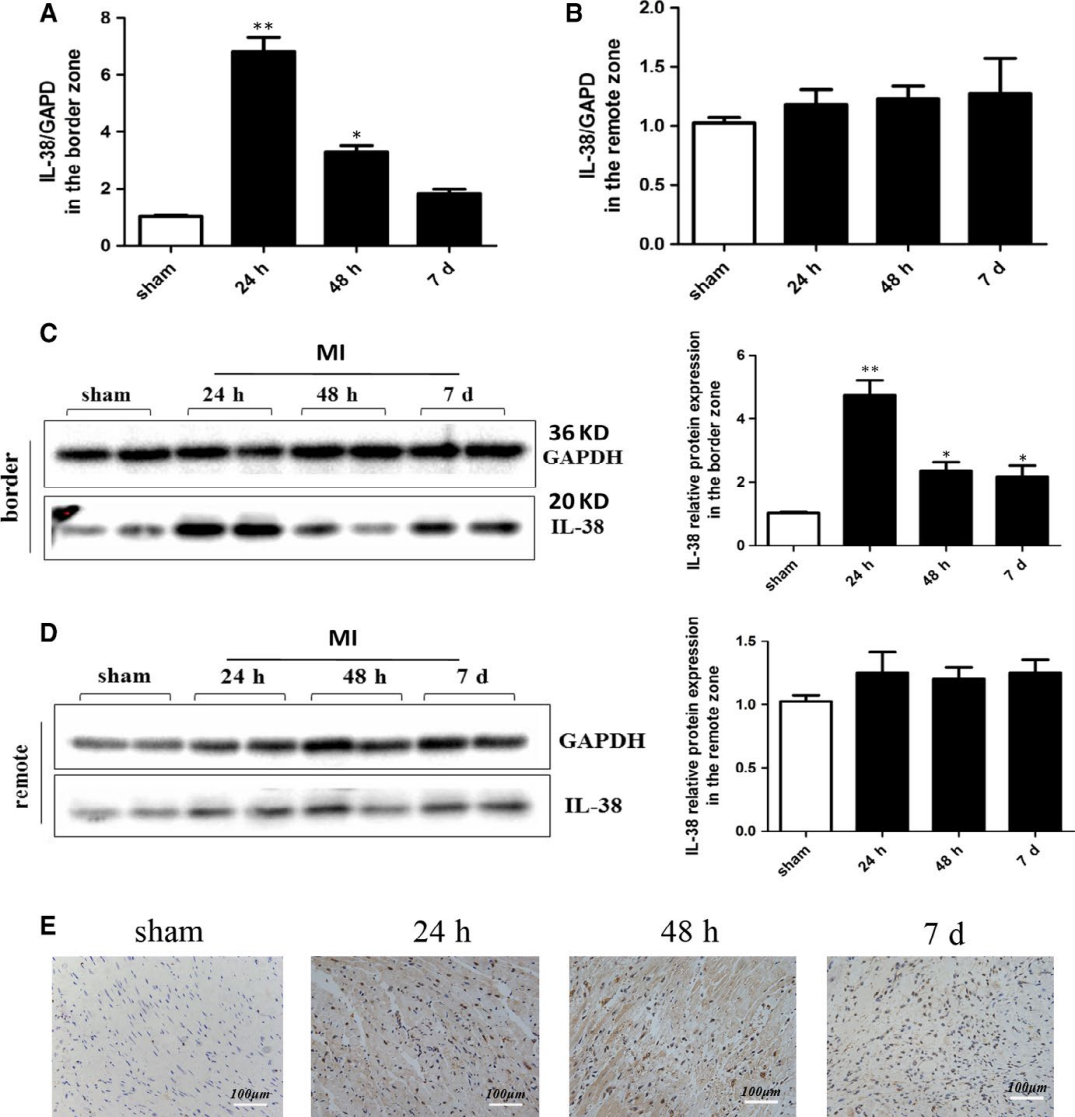
IL‐38 was increased in mice after MI. IL‐38 mRNA and protein expression levels were assessed by real‐time PCR and Western blot, respectively. Relative mRNA expression of IL‐38 in the border zone (A) and remote zone (B) of infarcted hearts in C57BL/6 at different time points post‐MI. Representative image of western blot for IL‐38 protein expression levels in the border zone (C) and remote zone (D) of infarcted hearts in C57BL/6 at different time points post‐MI. E, IL‐38 expression was reflected by immunohistochemical staining with IL‐38 antibody in infarcted heart at different time points post‐MI. (sham n = 6, 24 h n = 8, 48 h n = 8, 7 d n = 8).*P < .05 vs sham; **P < .01 vs sham

IL‐38 expression has been found in skin, tonsil, thymus, spleen, foetal liver and salivary glands,^23^ but very few data are available to concerning the expression level of IL‐38 in heart and the specific role of IL‐38 in cardiac inflammatory diseases. Firstly, we detected the expression level of IL‐38 in heart tissue and compared the expression of IL‐38 in the border zone and in the remote zone of infarcted heart in mice. As shown in Figure1A,B, the mRNA expression of IL‐38 was found to be increased obviously in the border zone of infarcted heart and peaked at 24 hours, but we did not find statistical significance in the remote zone of infarcted heart at different time‐points post‐MI. In addition, the protein expression level of IL‐38 in infarcted heart was consistent with the previous result of mRNA (Figure1C,D). IL‐38 level was increased after MI reflected by immunohistochemical staining with IL‐38 antibody in Figure 1E.

Our data indicate that endogenous IL‐38 in mice heart was increased during the resolution of acute inflammation of MI, suggesting that IL‐38 may act naturally as an important cytokine in the process of MI.

### 3.2 Cardiomyocytes are responsible for producing IL‐38

**Figure 2:**
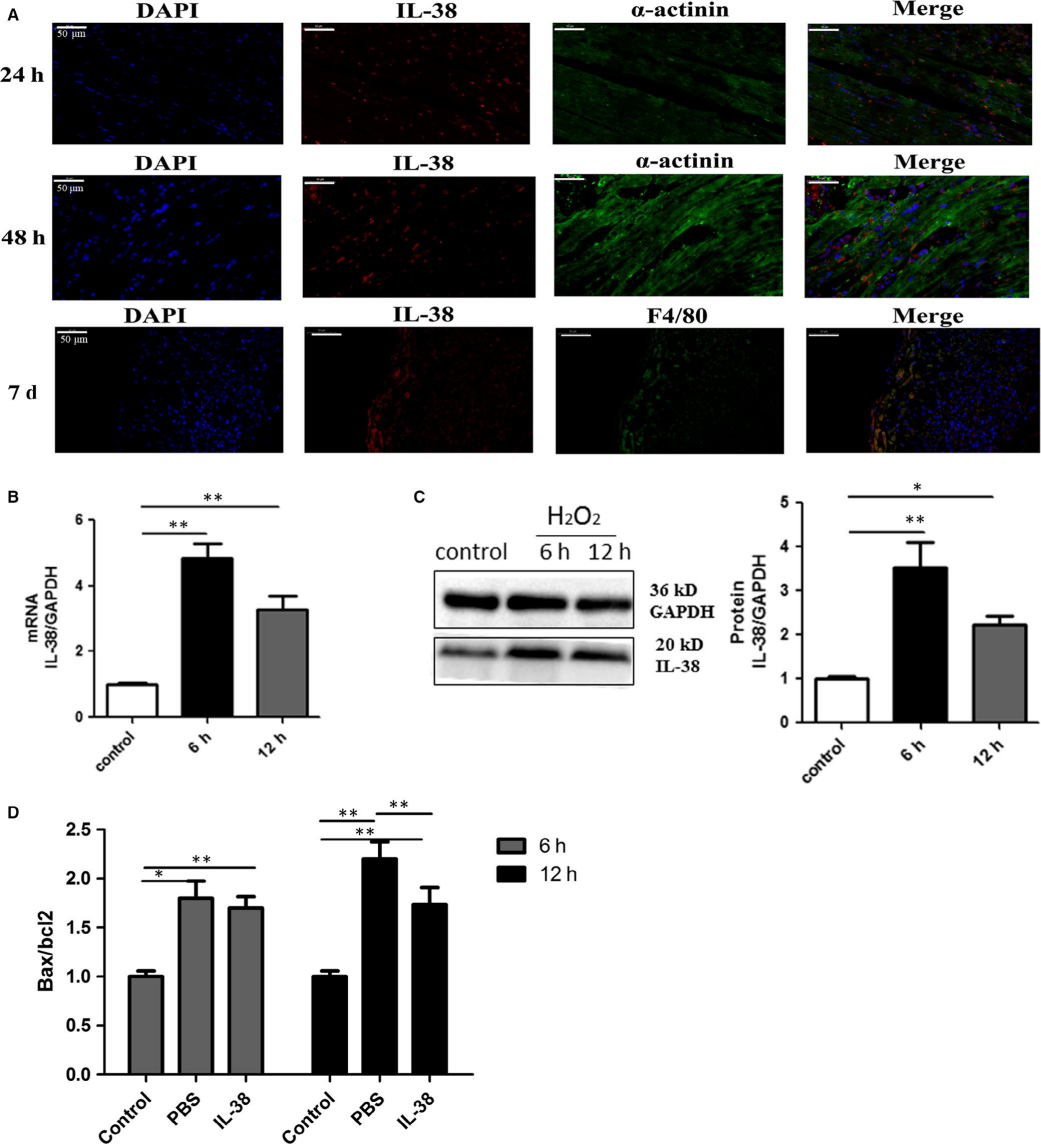
Cardiomyocytes are responsible for producing IL‐38 in infarcted heart. A, Representative images of infarcted heart sections stained with antibodies against cardiomyocyte marker α‐actinin (green), macrophage marker CD68 (green), and the cytokine IL‐38(red), and a nuclear stain (blue). Scar bar: 50 μm (each time point n = 8). B, IL‐38 mRNA expression levels in cultured cardiomyocytes with H_2_O_2_ stimulation for 6h and 12 h. C, IL‐38 protein expression levels in cultured cardiomyocytes with H_2_O_2_ stimulation for 6 and 12 h (control n = 6, 6 h n = 8, 12 h n = 8). D, Real‐time analysis of Bcl‐2 and Bax mRNA levels after incubation with rIL‐38 (50 ng/mL) for the indicated times. The results are expressed as the Bax/Bcl‐2 ratio (control n = 6, 6 h n = 8, 12 h n = 8). *P < .05 vs sham; **P < .01 vs sham

To determine which cells are the primary source of the production of IL‐38 in MI, we performed double immunofluorescence on the major cell types involved in myocardial infarct development. We set up different time‐points and discovered that IL‐38 was mainly expressed in cardiomyocytes at 24 and 48 hours and was even detected in CD68^+^ macrophages at 7 days after MI (Figure 2A). To further demonstrate that cardiomyocytes are the mainly cellular sources of IL‐38, we used hydrogen peroxide (H_2_O_2_) to stimulate freshly isolated neonatal rat cardiomyocytes. As previously observed in infarcted heart, low mRNA and protein levels of IL‐38 were detected in cardiomyocytes without any stimulation. Interestingly, after treatment with suitable concentration of H_2_O_2_, intracellular level of IL‐38 was increased whether for a 6‐hour stimulus or a 12‐hour stimulus (Figure 2B,C). In summary, these results suggested that inflamed cardiomyocytes are the mainly cellular sources of IL‐38 post‐MI and macrophages can also secrete IL‐38 in the late stage of MI. To further verify whether Il‐38 can directly affect cardiomyocytes, cardiomyocytes were stimulated with suitable concentration of H_2_O_2_ and cultured for a specified time in the presence or absence of rIL‐38 (50 ng/mL). As shown in Figure2D, IL‐38 can reduce the proto anti‐apoptotic ratio of Bcl‐2 family proteins.

### 3.3 The role of IL‐38 on survival and cardiac function

**Figure 3:**
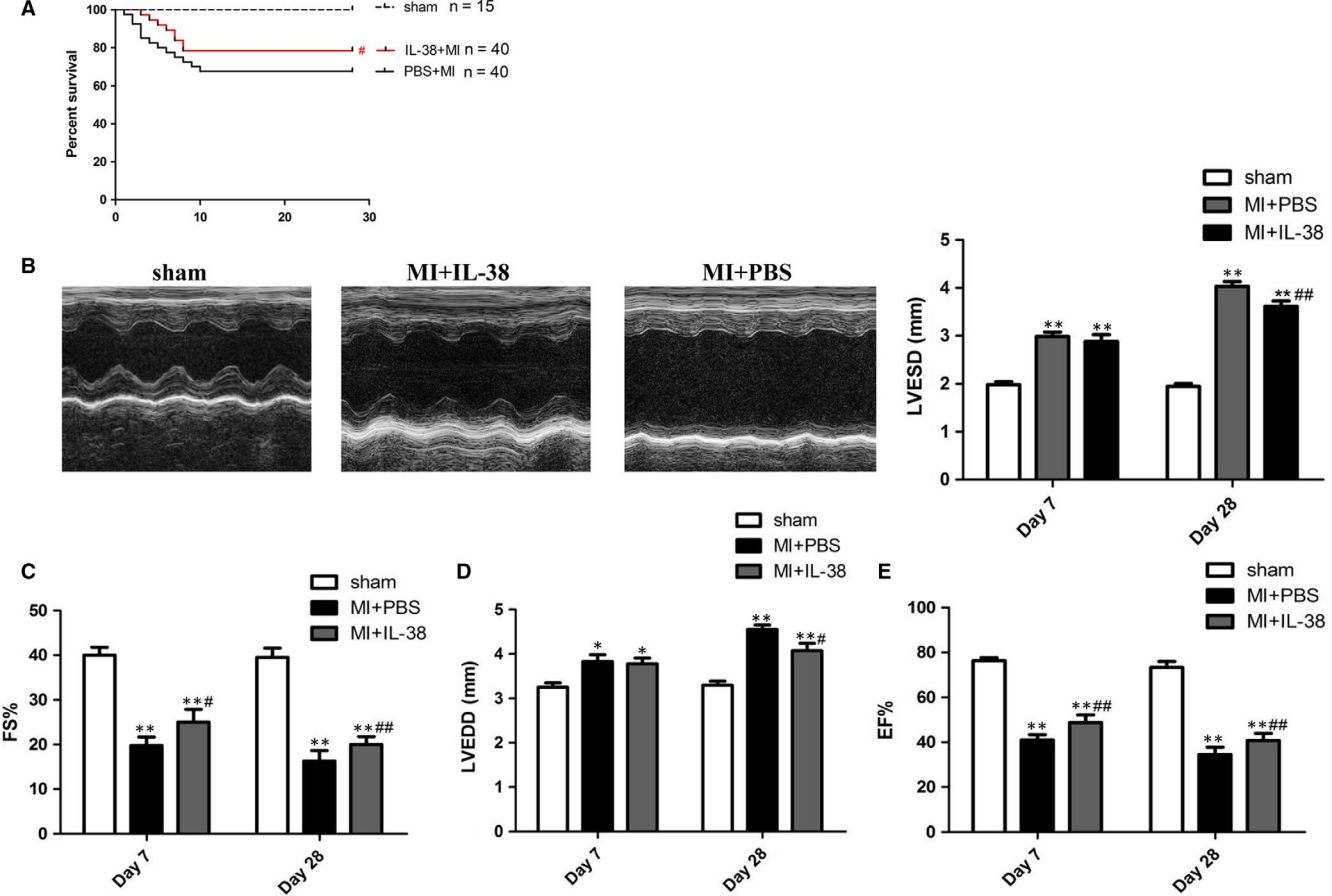
IL‐38 improves left ventricular function post‐MI. A, Survival analysis in PBS‐treated and IL‐38‐treated mice after MI within 28 days (sham n= 15, MI+PBS n=40 and MI+IL‐38 n = 40). B, Representative M‐mode echocardiography images of the left ventricle on day 28 post‐MI in different groups. C‐F, Ejection fraction (EF), ventricular end‐diastolic diameter (LVEDD), left ventricular end‐systolic diameter (LVESD) and fractional shortening (FS) on day 7 and day 28 post‐MI (sham n = 6, MI+PBS n = 8 and MI+IL‐38 n = 8). *P < .05 vs sham; **P < .01 vs sham; ^#^ *P* < .05 vs MI+PBS; ^##^ *P* < .01 vs MI+PBS

The effect of IL‐38 in mice with MI was determined by recombinant mouse IL‐38. The survival rate on day 28 post‐MI was 80% (32/40) in IL‐38‐treated mice and 65% (26/40) in PBS‐treated mice, indicating that IL‐38 decreased post‐MI mortality (Figure 3A).Cardiac dysfunction and ventricular dilation happened on day 7 post‐MI, altered rapidly at two weeks, and then changed slowly from one month.^24^ To compare cardiac function after MI in different groups, we calculated LVEDD, LVESD, EF and FS by echocardiography on day 7 and day 28 post‐MI. As shown in Figure 3B‐F, compared with PBS‐treated mice, echocardiographic assessment consistently revealed that EF and FS were greater in IL‐38‐treated mice on day 7 and day 28 post‐MI, LVEDD and LVESD was smaller on day 28 after MI. Thus, it seems that cardiac function was significantly prevented in the IL‐38‐treated group after MI.

### 3.4 IL‐38 inhibits cardiomyocyte apoptosis and alleviates cardiac fibrosis

**Figure 4:**
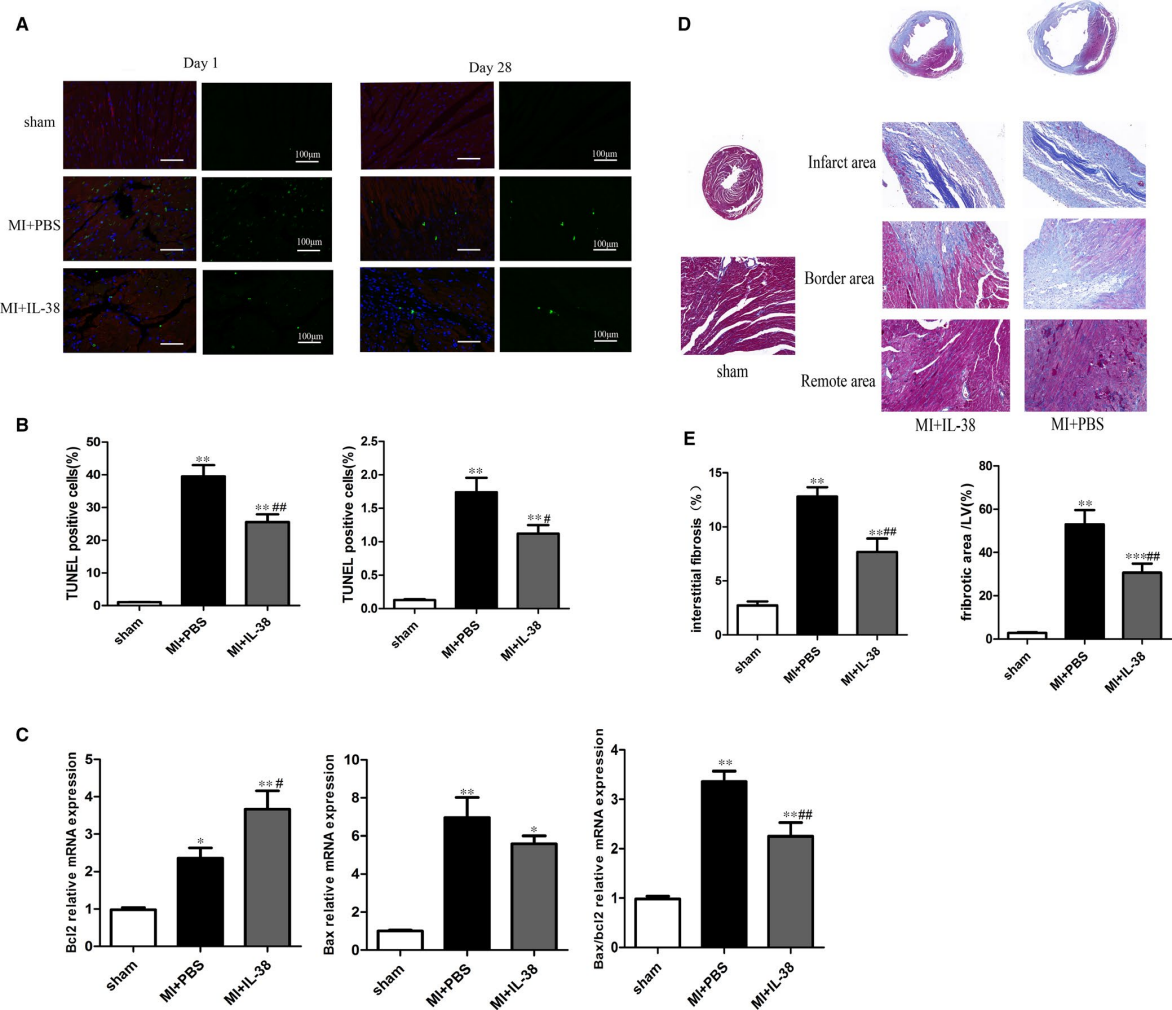
IL‐38 inhibited cardiomyocyte apoptosis and cardiac fibrosis in vivo. A, Representative images of terminal deoxynucleotidyl transferase dUTP nick‐end labeling (TUNEL)‐stained heart sections from different experimental groups 1 and 28 d post‐MI. TUNEL (green) and 4, 6‐diamidino‐2‐phenylindole (blue) staining of nuclei in apoptotic cardiomyocytes (red) in the peri‐infarct zone. Scar bar: 100 μm. B, Quantitative analysis of the percentages of TUNEL‐positive nuclei (each group n = 6). C, Real‐time PCR determined mRNA expression levels of Bax and Bcl‐2 in the in infarcted heart on day 1 after MI. The results were also expressed as ratio of Bax/Bcl‐2 (n = 4). D, Representative Masson’s trichrome staining images of collagen deposition (blue) in both infarct and remote areas on day7 post‐MI. E, The extent of fibrosis, as assessed by the fibrotic area/left ventricle and interstitial fibrosis, was compared among the different groups (each group n = 6). **P < .01 vs sham; ^#^ *P* < .05 vs MI+PBS; ^##^ *P* < .01 vs MI+PBS

IL‐38 has been shown to promote heart function in post‐MI mice, we next tried to determine the mechanism underlying how IL‐38 prevents the development of left ventricular remodelling. We first measured the number of apoptotic cells by TUNEL staining on days 1 and 28 after MI. As shown in Figure 4A,B, compared with PBS‐ treated group, rIL‐38 markedly decreased the number of TUNEL‐positive cardiomyocytes, which indicated that IL‐38 has an anti‐apoptotic effect on cardiomyocytes in both early and late stages post‐MI. In addition, mRNA levels of the expression of pro‐apoptotic molecules Bax was lower in IL‐38‐treated mice than in controls. In contrast, the expression of anti‐apoptotic molecule Bcl‐2 was higher in IL‐38‐treated mice than in controls (Figure 4C).

Dead myocardium is replaced by non‐contractile fibrous scar tissue that leads to ventricular dysfunction.^24^ We therefore analysed fibrosis on day 7 in the heart. As shown in Figure 4D,E, IL‐38‐treated mice exhibited markedly reduced fibrotic areas than PBS‐treated mice.

### 3.5 IL‐38 inhibits inflammatory response in infarcted heart

**Figure 5:**
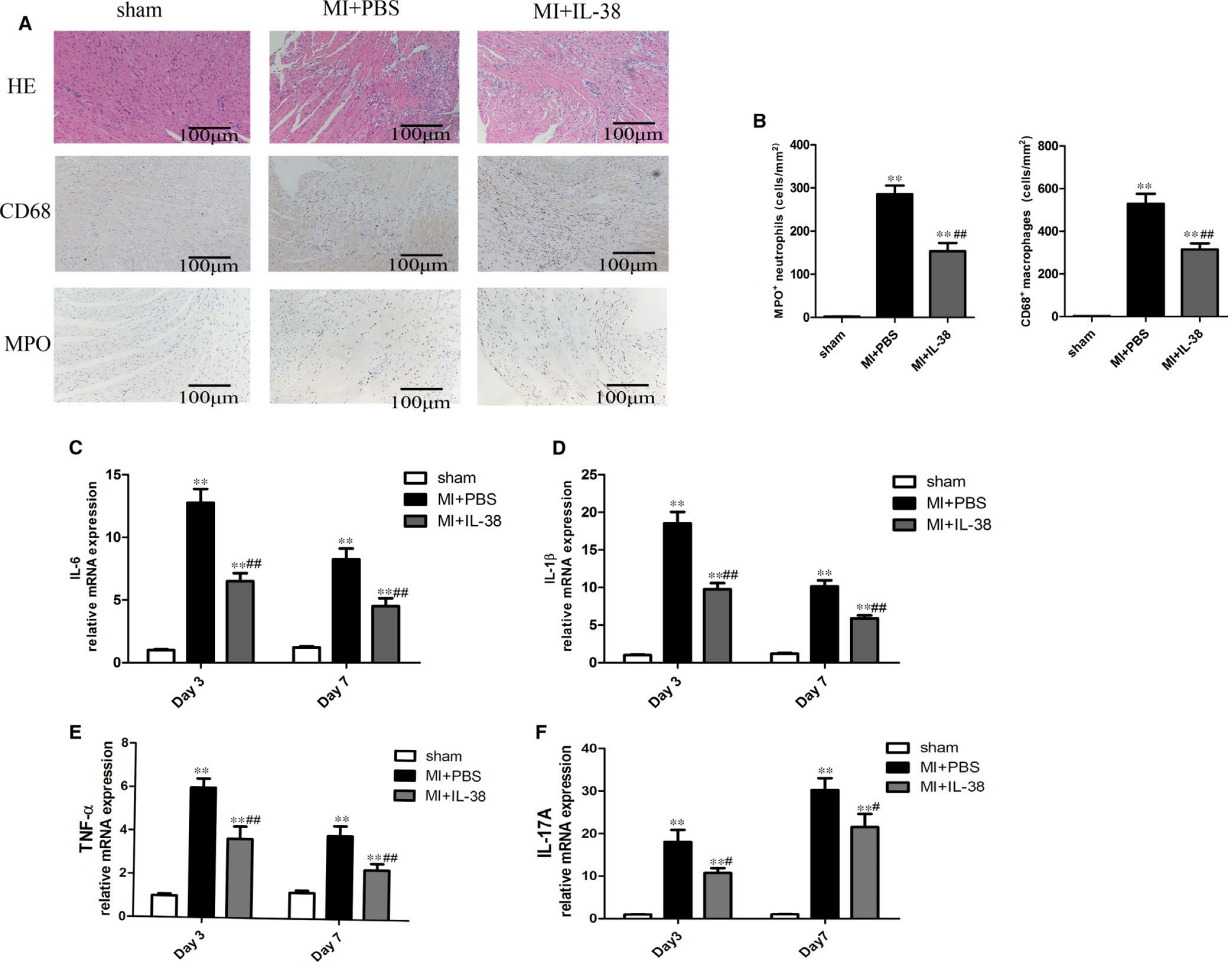
IL‐38 inhibits inflammatory response in the infarcted heart. A, Representative images of haematoxylin and eosin (HE) staining, infiltration of myeloperoxidase (MPO^+^) neutrophils and mouse CD68^+^ macrophages in the border area of infarcted hearts. Images for HE staining and neutrophils are from day 3 after MI, and images for macrophages are from day7 post‐MI. B, Infiltration of neutrophils and macrophages were compared between the different groups at set time points (each group n = 6). C–F, Analysis of mRNA levels of IL‐6, IL‐1β, TNF‐α and IL‐17A on day 3 and 7 after MI. Data are depicted as fold changes vs sham and shown as the mean ± SEM of 3‐6 independent experiments. **P < .01 vs sham; ^#^ *P* < 0.05^.^vs MI+PBS; ^##^ *P* < .01 vs MI+PBS

In the previous steps of the experiment, we found that IL‐38‐treated mice showed less cardiomyocyte apoptosis and cardiac fibrosis, so we tried to investigate whether the beneficial effects of IL‐38 are associated with the suppressed inflammatory responses. As shown in Figure 5A,B, infiltration of inflammatory cells was softer in IL‐38‐treated mice compared with PBS‐treated mice reflected by immunohistochemical staining with HE on day 3. Neutrophils are rapidly recruited in the event of heart damage, followed by proinflammatory monocytes and lymphocytes.^8^ We next tested infiltration of neutrophils on day 3 and macrophages on day 7 in damaged hearts. Data showed that the number of neutrophils that infiltrated into the infarcted myocardium was obviously lower in IL‐38‐treated mice than that in control mice, and the difference of macrophages infiltration degrees between the two groups was in line with this result (Figure 5A,B).

In the complex environment of infarcted heart, malignant inflammatory response may be somewhat prolonged due to the effects of a variety of cytokines. We next measured the spatially and temporally expression of cytokines in the infarcted heart. As shown in Figure 5C‐F, the levels of IL‐6, tumour necrosis factor (TNF)‐α, IL‐1β and IL‐17A were reduced in IL‐38‐treated mice than in control mice with MI.

### 3.6 The role of IL‐38 on the phenotype of DCs

**Figure 6:**
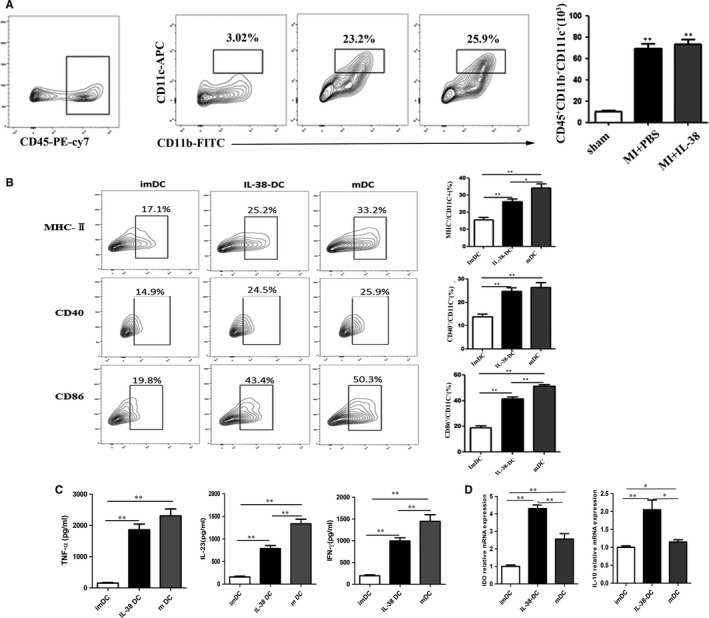
The role of IL‐38 on DCs. Bone marrow‐derived DCs were cultured in the absence of stimulus (imDCs) or in the presence of 100 ng/mL LPS (mDCs) or/and 100 ng/mL LPS plus 50 ng/mL IL‐38. DCs were stained with specific antibodies against major histocompatibility complex class II (MHC‐II), CD40 and CD86 and analyzed by flow cytometry. A, Comparison of the CD45^+^CD11b^+^CD11c^+^ cells in infarcted heart. B, Comparison of the CD11c^+^MHC‐II^+^, CD11c^+^CD40^+^ and CD11c^+^CD86^+^cells in different cultured DCs. C, TNF‐α, IL‐23 and IFN‐γ expression levels were assessed by ELISA in cultured supernatants of different DCs. D, IDO and IL‐10 mRNA expressions in cultured DCs.( each group n = 4 and repeat at least three times).*P < .05, **P < .01 vs sham

DCs act as an effective immune regulator during the post‐infarction healing process via its regulation of immune cells homeostasis.^25^ In addition, high expression of IL‐36R, the membrane receptor for IL‐36, IL‐36Ra and IL‐38, was detected on DCs.^14^ These reach results prompted us to investigate whether IL‐38 acts on DCs and therefore plays a further role on T lymphocytes. We first examined the infiltration of DCs into the heart after MI in different groups. Data analysed by Flowjo showed that the number of CD11c^+^ DC was increased in mice heart after MI. However, no obvious statistical significance was found between PBS‐treated and IL‐38‐treated mice with MI (Figure 6A). Next, we investigated the role of IL‐38 on the phenotype of bone marrow‐derived DCs generated with GM‐CSF and IL‐4 in vitro. As shown in Figure 6B, untreated DCs expressed low levels of major histocompatibility complex class II (MHC‐II), CD40 and CD86, and further stimulation with LPS increased expression of MHC‐II, CD40 and CD86, a characteristic of mature DCs. In addition, imDCs exposed to LPS and IL‐38 express lower levels of CD40 and CD86 compared with mDCs, although there was no difference in the expression levels of MHC‐II. The phenotype observed in the case of IL‐38‐DCs seemed to be characteristic of semimature DCs.

We next quantified TNF‐α, IFN‐γ and IL‐23in the supernatants of different cultured cells. We found that LPS‐DCs produced high amounts of TNF‐α, IFN‐γ and IL‐23, whereas imDCs and IL‐38‐DCs produced lower amounts of TNF‐α, IFN‐γ and IL‐23. Additionally, LPS‐DCs treated with IL‐38 inhibited the expression levels of TNF‐α, IFN‐γ and IL‐23(Figure 6C,D). Although the total number of DCs did not differ significantly between PBS‐treated and IL‐38‐treated mice with MI**,** the expression of molecules on the surface of cells in vitro was affected. Our results indicate that IL‐38 could target DCs during the post‐MI remodelling mainly by affecting the regulating function of DCs, rather than by affecting the amount of DCs infiltration.

### 3.7 IL‐38 plus TNI‐treated DCs exhibit more tolerogenic properties

**Figure 7:**
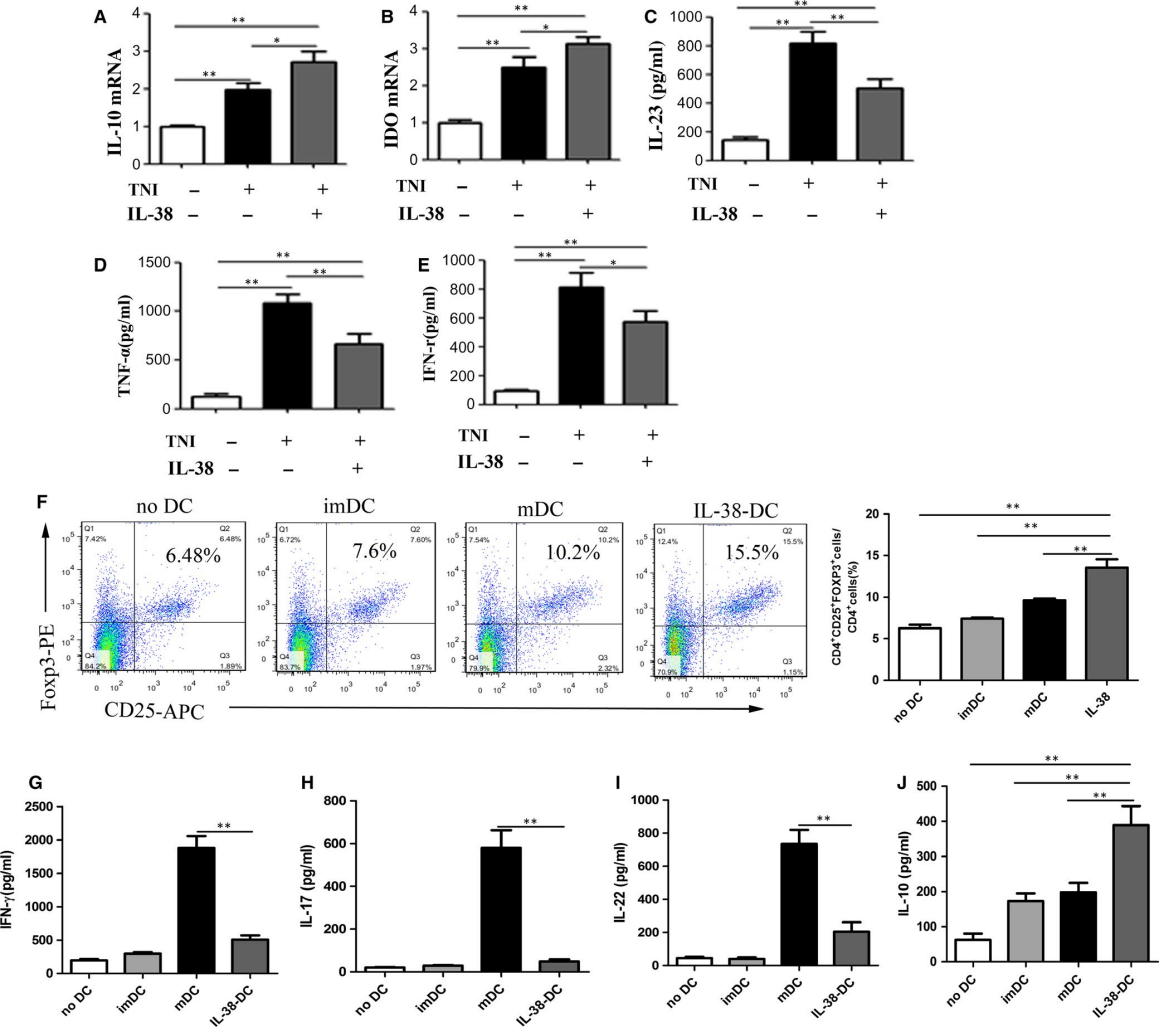
IL‐38 plus TNI treated DCs augment the percentage of regulatory T cells in vitro. We used small doses of LPS (10 ng/mL) and TNI (1 μg/mL) to induce the tolerance of DCs. A‐B, IL‐10 and IDO mRNA expression levels in cultured DCs. C‐E, IL‐23, TNF‐α, and IFN‐γ expression levels were assessed by ELISA in cultured supernatants of different DCs. F, Splenic CD4^+^ T cells (1 × 10^6^ cells/mL) were cultured for 3 d in the presence of medium alone or combined with imDCs, mDCs, or tDCs (2 × 10^5^ cells/mL). The cells were stained with anti‐CD4, anti‐CD25, and anti‐Foxp3 and analyzed by flow cytometry. G‐J, Analysis of the expression levels of IFN‐γ, IL‐17A, IL‐22 and IL‐10 in culture supernatants of CD4^+^ T cells using ELISA in different groups. (each group n=4 and repeat at least three times). *P < .05,**P < .01

Cardiac troponin I (cTNI) is a gold standard biomarker for the acute coronary syndrome, as it indicates myocardial cell damage with high sensitivity and specificity.^26^ TNI‐pulsed DCs can induce antigen‐specific immune responses. We used low‐dose LPS and TNI for inducing antigen‐specific tolerogenic DCs (tDCs). As shown in Figure 7A‐E, DCs pulsed with IL‐38 and TNI showed higher expressions levels of IDO and IL‐10 and lower expression levels of IL‐23, TNF‐α and IFN‐γ than TNI‐loaded DCs.

### 3.8 IL‐38‐tDCs augment the percentage of regulatory T cells in vitro

CD4^+^ T cell activation and homeostasis are involved in facilitating wound healing after MI.^27^ Next, a series of functional co‐culture experiments were performed to investigate the role of IL‐38‐treated tDCs on CD4^+^T cells. As shown in Figure 7F, IL‐38‐tDCs increased the percentage of Foxp3^+^Treg cells when co‐cultured with CD4^+^ T, compared with mDCs and imDCs. We then quantified IL‐17, IL‐22, IFN‐γ and IL‐10 in the supernatants of different co‐cultured cells. As shown in Figure 7G‐I, IL‐38‐tDCs produced lower amounts of IL‐17, IL‐22, IFN‐γ than LPS‐DCs; on the contrary, cultures with IL‐38‐tDCs induced the secretion of IL‐10 (Figure 7J). These findings suggested that IL‐38‐tDCs acquired the capacity to induce tolerogenic conditions when co‐cultured with CD4^+^T cells.

## 4 Discussion

Previously, our clinical data indicated that circulating IL‐38 is a potentially novel biomarker for in STEMI patients.^20^ In the present study, we further investigated the role of rIL‐38 in ventricular remodelling after MI. We found that MI induced IL‐38 expression and rIL‐38‐treated mice showed better cardiac function and less myocardial injury than PBS‐treated mice after MI. In addition, IL‐38 inhibited DCs maturation and IL‐38‐ tDCs acquired the capacity to induce tolerogenic conditions when co‐cultured with CD4^+^T cells. Taken together, these findings identify IL‐38 as a potential therapeutic target in post‐MI cardiac remodelling.

Myocardial necrosis can trigger an inflammatory response to remove dead cells and matrix debris from the site of injury, which can be beneficial for activating reparative pathways.^7^, ^8^ However, accentuation or prolongation of the post‐infarction inflammatory response ultimately exacerbates tissue injury and results in worse cardiac remodelling and dysfunction following MI.^8^, ^28^, ^29^ IL‐38 is the most recently identified cytokine of IL‐1 family and genetic association studies indicate that IL‐38 might be involved in human inflammatory diseases.^30^, ^31^ Polygenetic analysis has reported that rs6734238 in the *IL1F10/IL1RN* locus was found to be significantly associated with coronary artery disease.^32^ Additionally, in atherosclerotic plaques of patients with coronary heart disease (CAD), Jha HC et al found that expression level of IL‐38 was significantly increased in cHSP60 negative patients compared with the cHSP60‐positive group.^33^ We previously reported that circulating IL‐38 was increased in STEMI patients, which indicated that IL‐38 may act as a promotive role in the development of MI.^20^ In this study, we observed that administration of rIL‐38 in mice ameliorated ventricular remodelling via suppressing infiltration of inflammatory cells and inhibiting proinflammatory cytokines in the post‐MI hearts. Therefore, high IL‐38 levels in patients with STEMI may represent an adaptive mechanism aimed at preventing the progression of harmful ventricular remodelling after MI. Overall, our results were in accordance with prior studies showing that effectively restricting the excessive acute inflammatory response can improve cardiac function via attenuating ventricular remodelling.^10^, ^34^, ^35^

IL‐38 expression has been reported in many organs and tissues, such as skin, tonsil, thymus, spleen, foetal liver and salivary glands.^36^ It has been recently found that IL‐38 can be released from apoptotic cells to limit inflammatory response induced by macrophage.^19^ However, few reports of its expression or function in heart disease have been reported. Whether intrinsic cardiac cells or circulating and/or homing extracardiac cells were responsible for producing IL‐38 was not known. In this study, we discovered that IL‐38 was increased, especially in peri‐infarct zone of mice heart with MI. IL‐38 was mainly expressed in cardiomyocytes in the process of MI, though it was even detected in CD68^+^ lesional macrophages at 7 days after MI. We also found a similar effect in cultured cardiomyocytes exposed to exogenous H_2_O_2_ oxidative stress. Overexpression of IL‐38 in cultured cardiomyocytes reduced the proto anti‐apoptotic ratio of Bcl‐2 family proteins, which is an upstream regulator of mitochondrial cytochrome c release. The ratio of Bax to Bcl‐2 is an important determinant of the cell’s susceptibility to undergo apoptosis.^37^ Our data indicated that inflamed cardiomyocytes are the mainly cellular sources of IL‐38 in post‐MI and IL‐38 may affect the apoptosis of cardiomyocytes by regulating Bcl2/Bax pathway.

We used rIL‐38 for determining the role of IL‐38 in MI mice. Our strategy of IL‐38 administration improved cardiac morphology and function as early as 1 week post‐MI. The decrease of inflammatory cell infiltration and proinflammatory cytokines expression suggest us IL‐38 may play a negative role in the inflammatory response after post‐MI. Therefore, we hypothesized that it could not only act directly on cardiomyocytes, but also can act on other inflammatory cells, thereby regulating the immune inflammatory response. It is well accepted that DCs include a heterogeneous family of professional antigen‐presenting cells (APCs) involved in initiation of immunity and immunologic tolerance. Immature DCs and partially or semimature DCs are tolerogenic, whereas fully mature DCs are immunogenic.^38^, ^39^ Normally, tolerogenic DCs play critical roles in inducing peripheral tolerance by suppressing effector T cells, activating regulatory (Treg) cells and negative modulating Th1/Th2 immune responses.^40^, ^41^ Interestingly, Toshihisa et al have reported that DCs act as a potent immune protective regulator via its control of monocyte/macrophage homeostasis in the post‐infarction healing process.^25^ In addition, high expression of IL‐36R, the membrane receptor for IL‐36, IL‐36Ra and IL‐38, was detected on DCs.^14^ We therefore speculated that the anti‐inflammatory role of IL‐38 in post‐MI remodelling was involved in regulation of DCs. As speculated, we found that IL‐38 inhibited DCs maturation induced by LPS, characterized by lower expression levels of cell surface molecule and inflammatory factors than LPS‐DCs.

Additionally, it has been demonstrated that superabundant of effective T cells and insufficient recruitment of Treg cells results in exacerbating ventricular remodelling after MI.^10^, ^42^ Tregs may suppress inflammation via the control of secreting inhibitory signals such as IL‐10 and transforming growth factor (TGF)‐ β.^43^ Andreas Hermansson et al have stated that ApoB100‐Loaded tolerogenic DCs have the potential to induce antigen‐specific CD4^+^ Treg cells that affect activation of a T‐cell hybridoma.^44^ The ability of IL‐37‐DCs in activating naïve T cells and inducing Treg cells has also been reported.^11^, ^45^ In the present study, IL‐38 combined with TNI‐induced tDCs has stronger tolerance function compared with TNI‐induced tDCs, which demonstrated by higher mRNA expressions of IDO and IL‐10 and lower expression levels of IL‐23,TNF‐α and IFN‐γ in different DCs cultured supernatants. In addition, IL‐38‐tDCs markedly increased the percentage of Foxp3^+^Treg cells when co‐cultured with CD4^+^ cells. In vivo, though we found no significant difference in the number of cardiac DCs between PBS‐treated and rIL‐38‐treated mice with MI, rIL‐38 significantly reduced the levels of inflammatory cytokines which are related to DCs and CD4^+^ cells. Data in vitro and vivo indicated that IL‐38 could target DCs during the post‐MI remodelling mainly by affecting the regulating function of DCs, rather than by affecting the amount of DCs infiltration. However, adoptive transfer of rIL‐38‐DCs into mice is lacking. We still need further detailed study.

So far, the receptors for IL‐38 have not been fully established. IL‐38 might act as an IL‐1 family antagonist for the highly homologous toIL‐36Ra and IL‐1Ra.^31^ It has been shown that IL‐38 binds to IL‐36R and neutralizes the IL‐36 cytokine signalling to exert anti‐inflammatory effects.^14^ However, recently study reported that IL‐38 can bind toIL‐1RAPL1 to limit cytokine production in a broader inflammatory context.^19^ Here, we showed that IL‐38 can exert anti‐inflammatory effects post‐MI via affecting the phenotype of DC, suggesting that IL‐38 could target DCs in addition to macrophages. However, according to the present results, it is difficult to distinguish the underlying mechanism for the beneficial effect of IL‐38. We need IL‐38 knockout mice and specific DCs knockout mice for further mechanism study. Therefore, the molecular mode of action and receptor signalling pathway of IL‐38 in MI need to be further investigated.

In conclusion, we demonstrated that IL‐38 plays a protective effect in ventricular remodelling post‐MI in this study, one possibility by influencing the regulatory function of DCs to attenuate inflammatory response. However, relatively small sample size and unclear signal mechanism of IL‐38 should be considered in the study. Whether IL‐38 should be considered as a new therapeutic molecule in MI deserves further experiments.

## Conflict of Interest

None declared.

## Author contribution

Conception and design: Qiutang Zeng ,Yuzhen Wei , Yin Lan, Yucheng Zhong. Acquisition, analysis, or interpretation: Yuzhen Wei, Yin Lan, Yucheng Zhong, Kunwu Yu, Wenbin Xu, Ruirui Zhu, Haitao Sun, Yan Ding, Yue Wang. Drafted the manuscript : Yuzhen Wei. All authors have read and approved the final manuscript.

## Data Availability

The data can be available and cited.
